# Extracorporeal Shock Wave Therapy Versus Local Corticosteroid Injection for Chronic Lateral Epicondylitis: A Systematic Review with Meta‐Analysis of Randomized Controlled Trials

**DOI:** 10.1111/os.14212

**Published:** 2024-08-28

**Authors:** Lei Zhang, Xinyi Zhang, Long Pang, Zhuo Wang, Junliang Jiang

**Affiliations:** ^1^ Rehabilitation Medicine Center and Institute of Rehabilitation Medicine, West China Hospital Sichuan University Chengdu China; ^2^ Key Laboratory of Rehabilitation Medicine in Sichuan Province, West China Hospital Sichuan University Chengdu China; ^3^ College of Medical, Veterinary and Life Sciences University of Glasgow Glasgow UK; ^4^ Sports Medicine Center, West China Hospital Sichuan University Chengdu China; ^5^ Department of Orthopedics, Orthopedic Research Institute, West China Hospital Sichuan University Chengdu China

**Keywords:** Extracorporeal Shock Wave Therapy, Lateral Epicondylitis, Local Corticosteroid Injection, Meta‐Analysis, Systematic Review

## Abstract

Chronic lateral epicondylitis (LE), normally known as tennis elbow, is often managed by conservative treatments. Extracorporeal shock wave therapy (ESWT) and local corticosteroid injection (LCI) are among the most commonly used conservative treatments. However, the comparison between these two interventions remains controversial. This study aimed to compare the effectiveness and safety of ESWT and LCI for chronic LE. A systematic review and meta‐analysis was conducted following the Preferred Reporting Items for Systematic Review and Meta‐analyses (PRISMA) guidelines. PubMed, EMBASE, Cochrane Library, and Web of Science were searched for eligible studies until April 20, 2024. Meta‐analyses were conducted using Manager V.5.4.1. Pooled effect sizes were expressed as the weighted mean difference (WMD) or odds ratio (OR), with 95% confidence intervals (CIs). A total of six randomized controlled trials (RCTs) were included. Compared with LCI, ESWT had inferior change in visual analogue scale (Δ VAS) (WMD, 1.14; 95% CI, 0.80 to 1.48; *I* ^2^ = 20%; *p <* 0.001), Δ grip strength (WMD, −4.01; 95% CI, −5.57 to −2.44; *I* ^2^ = 36%; *p <* 0.001), change in patient‐rated tennis elbow evaluation (Δ PRTEE) score (WMD, 8.64; 95% CI, 4.70 to 12.58; *I* ^2^ = 0%; *p <* 0.001) at 1‐month follow‐up, but superior Δ VAS (WMD, −1.15; 95% CI, −1.51 to −0.80; *I* ^2^ = 6%; *p <* 0.001), Δ grip strength (WMD, 2.04; 95% CI, 0.90 to 3.18; *I* ^2^ = 3%; *p* = 0.0005), Δ PRTEE score (WMD, −9.50; 95% CI, −14.05 to −4.95; *I* ^2^ = 58%; *p <* 0.001) at 3‐month follow‐up, and superior Δ VAS (WMD, −1.81; 95% CI, −2.52 to −1.10; *I* ^2^ = 33%; *p <* 0.001), Δ grip strength (WMD, 3.06; 95% CI, 0.90 to 5.21; *I* ^2^ = 0%; *p* = 0.005) at 6‐month follow‐up. The two groups had a similarly low rate of adverse events (OR, 0.69; 95% CI, 0.05 to 8.60; *I* ^2^ = 67%; *p* = 0.77), all of which were mild. Both ESWT and LCI are effective and safe in treating chronic LE. Compared with LCI, ESWT showed inferior short‐term (1‐month) but superior long‐term (3‐month and 6‐month) outcomes regarding pain relief and function recovery, with a similar rate of mild adverse events.

## Graphical abstract

This systematic review and meta‐analysis of six randomized controlled trials (RCTs) compared the effectiveness of extracorporeal shock wave therapy (ESWT) *versus* local corticosteroid injection (LCI) for treating chronic LE. A total of 149 patients were treated with ESWT, and 127 patients received LCI. The analysis revealed that while ESWT showed less effective short‐term (1 month) outcomes for pain relief and functional improvement, it provided superior long‐term (3 and 6 months) benefits compared with LCI. Both treatments had similar rates of mild adverse events.

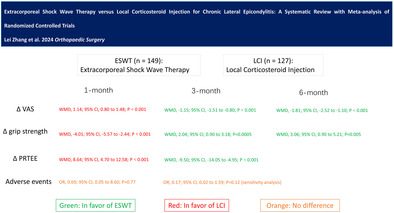

## Introduction

Lateral epicondylitis (LE), commonly known as tennis elbow, is believed to result from overuse or chronic degenerative changes at the origin of the extensor tendons in the elbow.^1^, ^2^ LE presents as lateral elbow pain, often accompanied by tenderness and occasional forearm weakness, typically without any discernible trauma.^2^ In population‐based research, LE showed an incidence of 3.4 cases per 1000 person‐years, with similar rates across genders.^3^ Other studies estimated a prevalence of 1%–3% among adults,^4^ peaking in the 40–49 age group, followed by those aged 50–59.^3^ Risk factors for LE include tobacco use, obesity, and high physical load, such as manual labor.^4^

Although the natural history of LE is not well characterized, conservative treatments including nonsteroidal anti‐inflammatory drugs (NSAIDs),^5^ splints or braces,^6^, ^7^ physical therapy,^8^ and injections^9^ using corticosteroids (CSs) or other agents such as botulinum toxin A (BoNT‐A), autologous whole blood (AWB), dextrose prolotherapy (DPT), or platelet‐rich plasma (PRP) remain the main options to manage LE.^2^, ^10^, ^11^ For chronic LE, extracorporeal shock wave therapy (ESWT) and local corticosteroid injection (LCI) are widely used.^12^ Although both open and arthroscopic surgical treatments for chronic LE have shown satisfying outcomes,^13^ surgery is only considered after conservative treatments have failed.

ESWT, noninvasive with a low risk of adverse effects, promotes soft‐tissue healing by immediately inhibiting pain receptors, reducing inflammatory cytokines, enhancing angiogenesis, and boosting cellular proliferation and extracellular matrix synthesis.^14^ However, it may necessitate multiple sessions and higher costs. Conversely, LCI offers rapid symptomatic relief, requiring fewer treatment sessions and lower costs. Nevertheless, its uncertain long‐term effectiveness and risks associated with repeated or high‐dose use make it remain a controversial option.^15^

Prior systematic reviews and meta‐analyses have yielded inconsistent findings regarding the comparison between ESWT and LCI. The limited number of studies with a high level of evidence (LOE) has significantly undermined the strength of their conclusions. With the emergence of new randomized controlled trials (RCTs), there is an urgent need for an updated systematic review with meta‐analysis of RCTs to offer the most current and highest LOE available.

This systematic review with meta‐analysis aims to compare the effectiveness and safety of ESWT and LCI in treating chronic LE. It is hypothesized that both two interventions are equally successful in treating chronic LE, but the incidence of complications would be different.

## Method

This systematic review with meta‐analysis adhered to the guidelines outlined in the Preferred Reporting Items for Systematic Review and Meta‐analyses (PRISMA) statement.^16^ Furthermore, the study was preregistered in The International Prospective Register of Systematic Reviews (PROSPERO) under the registration ID CRD42024506711.

### Search Strategy

Two independent reviewers conducted a comprehensive search on the PubMed, EMBASE, Cochrane Library, and Web of Science databases, including records up to April 20, 2024. The main search phrases utilized were as follows: (Extracorporeal shock wave therapy OR Shock wave therapy OR Shock wave OR ESWT) AND (Local corticosteroid injection OR Corticosteroid injection OR Steroid injection OR Corticosteroid OR Steroid) AND (Lateral epicondylitis OR Tennis elbow OR Lateral elbow tendinopathy). Any discordance encountered during the search was resolved through consultation with a third researcher.

### Selection Criteria

Inclusion criteria were as follows: (1) participants included were adults with a history of LE for at least 3 months; (2) RCTs; (3) ESWT and LCI were directly compared.

Exclusion criteria were as follows: (1) participants with any sign indicating dysfunction in the shoulder, neck, or thoracic region, localized arthritis, widespread polyarthritis, generalized neurological abnormalities, or entrapment of nerves in the upper limb; (2) participants with previous elbow fracture or dislocation; (3) participants with previous elbow surgeries; (4) participants with previous ESWT or LCI within 6 months; (5) not written in English.

### Data Extraction

Two researchers independently extracted data from the included studies, with any disagreements resolved by a third author. We communicated with the authors of the studies to obtain supplementary information as needed. The extracted information included the first author, year of publication, country where the study was conducted, LOE, sample size, patient demographic data (mean age, gender), symptom duration, follow‐up time points, and details of ESWT and LCI.

Primary outcomes were pain relief assessed by the change in visual analogue scale (VAS) scores (Δ VAS), and functional improvement assessed by the change in grip strength (Δ grip strength) at different follow‐up time points. Secondary outcomes were the change in patient‐rated tennis elbow evaluation (PRTEE) (Δ PRTEE) scores at different follow‐up time points, and adverse events. VAS is a widely used tool for assessing pain intensity, where patients rate their pain on a continuous line, typically ranging from 0 (no pain) to 10 (worst possible pain).^17^ Grip strength was measured using a hand‐held dynamometer, with patients exerting maximal force while gripping the device. Normally, the average of three trials was used for the analysis.^18^ PRTEE is a patient‐reported outcome measure specifically designed for assessing symptoms and function in individuals with LE. The PRTEE consists of a series of questions that cover pain, grip strength, and functional activities, with scores standardized to a 100‐point scale.^19^

### Quality Assessment

Two researchers independently assessed the methodological quality of the included studies using the revised Cochrane Risk of Bias 2 (RoB2) tool for RCTs.^20^ Kappa statistics was used to assess inter‐rater agreement (<0: less than chance agreement; 0.01–0.20: mild agreement; 0.21–0.40: reasonable agreement; 0.41–0.60: moderate agreement; 0.61–0.80: significant agreement; 0.81–0.99: almost perfect agreement). Any discrepancies among authors were resolved through thorough discussion and subsequent review by a third investigator. Assessment for publication bias was omitted due to the number of studies included in this field is less than 10, as recommended by the Cochrane Handbook.

### Statistical Analysis

Statistical evaluations were conducted using Manager V.5.4.1 (The Cochrane Collaboration, Software Update, Oxford, UK). We calculated the weighted mean difference (WMD) and pooled odds ratio (OR) with corresponding 95% confidence intervals (CIs) to assess continuous variables and dichotomous variables, respectively. Heterogeneity among studies was assessed using Cochrane's *Q* statistics and *I* ^2^ statistics, with *I* ^2^ < 50% considered acceptable heterogeneity and a fixed‐effect model applied. When *I* ^2^ > 50% or not applicable, a random‐effects model was applied. We adopted forest plots to visualize the pooled effect sizes. *p* values less than 0.05 were considered statistically significant.

## Results

### Characteristics of the included studies

**FIGURE 1:**
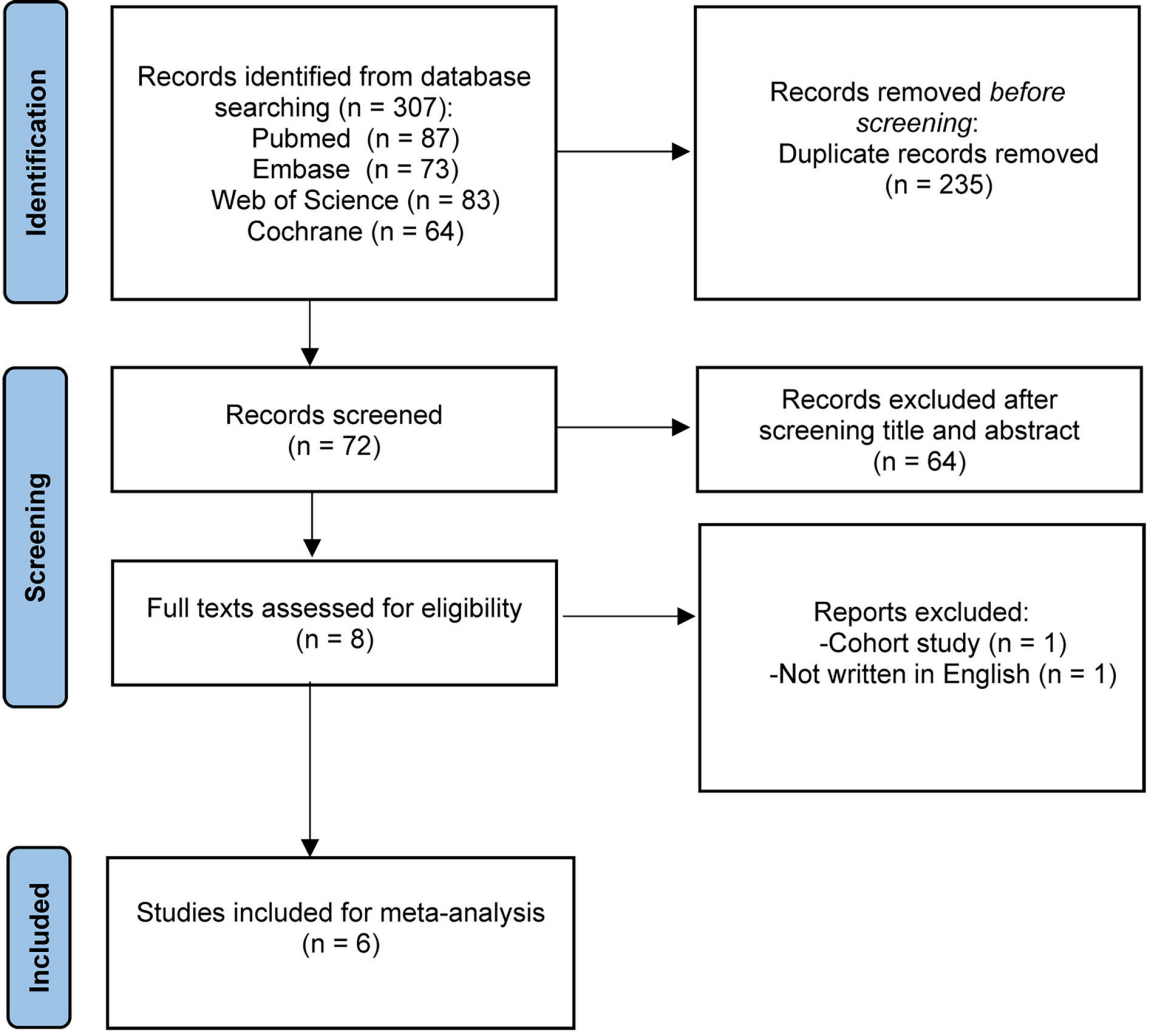
Flowchart of literature retrieval.

A total of 307 studies were initially identified through searches in PubMed, EMBASE, the Cochrane Library, and Web of Science. After removing 235 duplicate studies, the titles and abstracts of the remaining 72 publications were screened. Subsequently, 64 studies were further discarded, and the full texts and references of 8 articles were reviewed for eligibility. One cohort study and one study not written in English were excluded. Finally, six RCTs^21^, ^22^, ^23^, ^24^, ^25^, ^26^ were included (Figure 1). Table 1 outlines the characteristics of these included studies.

**TABLE 1:** Characteristics of the included studies

First author	Year	Country	LOE	Patients (elbows), n		Age, y Mean ± SD (Range)		Sex (M/F)		Symptom duration, m		Follow‐up time points, w	
				ESWT	LCI	ESWT	LCI	ESWT	LCI	ESWT	LCI	ESWT	LCI
Crowther	2002	UK	II	48 (48)	25 (25)	49 ± 10.5 (27–69)		25/23	13/12	≥4		6, 12	
ESWT	ESWT (2000 shocks maximum 0.1 MJ/mm^2^) with inline ultrasound guidance for three sessions (1‐week interval between sessions) using the portable Storz Minilith SL1 lithotripter.												
LCI	An injection of 20 mg of triamcinolone made up to 1.5 ml with 1% lignocaine using an aseptic technique, into the point of maximal tenderness at the extensor origin of the lateral epicondyle of the humerus.												
Ozturan	2010	Turkey	II	19 (19)	20 (20)	47 ± 8.7	45.8 ± 8.1	8/11	10/10	9.6 ± 2.7	9.5 ± 3.1	4, 12, 26, 52	
ESWT	ESWT (2000 shocks at 0.17 MJ/mm^2^) for three sessions (1‐week interval between sessions) using a shock wave tube (Stonelith V5 lithotriptor; PCK Electronic Industry & Trade Co, Ankara, Turkey).												
LCI	A local anesthetic injection (prilocaine 1 ml) to the skin and subcutaneous tissues, followed by methylprednisolone acetate (1 ml) injection with five skin penetrations at the tender part of the tendon, using one skin portal according to the technique of Mishra and Pavelko.^45^												
Gündüz	2012	Turkey	II	20 (20)	20 (20)	44.9 ± 9.9	45.7 ± 10.2	8/12	8/12	30 ± − (1–90)	30 ± − (2–90)	4, 12, 26	
ESWT	ESWT (pressure 1.4 bar, frequency 4.0 Hz, 500 shocks) for 10 sessions (1‐day interval between sessions).												
LCI	A single injection of 20 mg methylprednisolone acetate with 1 ml prilocaine.												
Beyazal	2015	Turkey	II	32 (32)	32 (32)	38.7 ± 9.1	42.6 ± 6.6	26/6	28/4	8.5 ± 3.5	7.9 ± 3.0	4, 12	
ESWT	ESWT (pressure 1.6 bar, frequency 16 Hz, 2000 shocks) for 3 sessions (1‐week interval between sessions) using a Masterpuls MP2004 radial shock wave therapy system (Storz Medical, Swiss).												
LCI	A single injection of 20 mg methylprednisolone acetate with 1 ml prilocaine using a peppering technique at the point with maximal tenderness in the lateral epicondyle area.												
Ismael	2020	Egypt	II	15 (15)	15 (15)	44.7 ± 6.1	46.0 ± 6.0	9/6	8/7	8.2 ± 2.9	8.0 ± 2.9	4, 12	
ESWT	ESWT (pressure 1.6 bar, frequency 16 Hz, 2000 shocks) for three sessions (1‐week interval between sessions).												
LCI	A single injection of 20 mg methylprednisolone acetate with 0.6 ml of lidocaine.												
Ibrahim	2021	Egypt	II	15 (15)	15 (15)	32.4 ± 6.3	31.0 ± 7.3	12/3	10/5	4.3 ± 2.3	3.9 ± 2.2	2, 4, 8, 12	
ESWT	Radical ESWT (pressure 1.2 bar, frequency 4 Hz, 500 shocks, 0.144 MJ/mm^2^) for three sessions (1‐week interval between sessions).												
LCI	A single injection of 40 mg methylprednisolone with 1 ml mepacaine at the point with utmost tenderness in the lateral epicondyle area.												

Abbreviations: ESWT, extracorporeal shock‐wave therapy; F, female; LCI, local corticosteroid injection; LOE, level of evidence; M, male; UK, the United Kingdom.

### Quality Assessment

**FIGURE 2:**
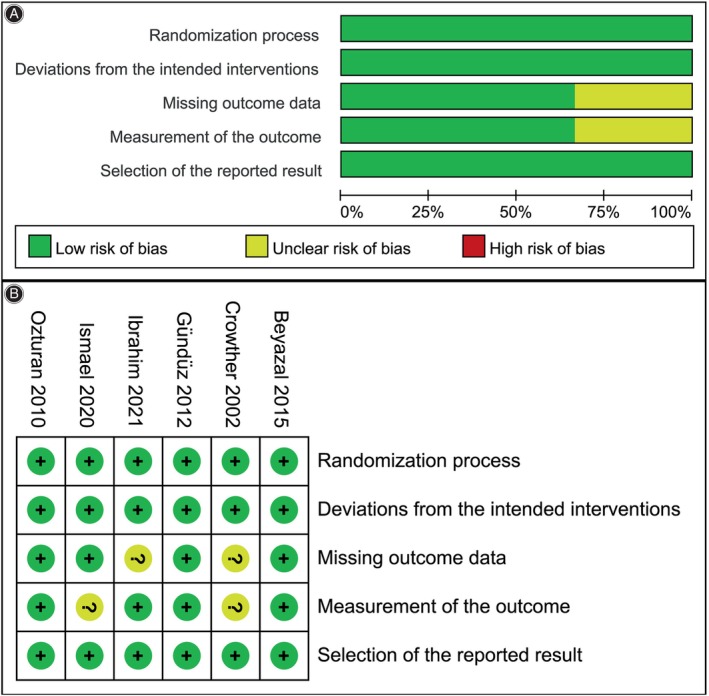
Risk‐of‐bias graph. (A) Graph of the risk‐of‐bias summary; (B) graph of the risk of bias for each included randomized controlled trial.

The quality assessment of the included RCTs used the revised RoB2 tool, as depicted in Figure 2. Inter‐rater agreement was excellent for the randomization process, deviations from the intended interventions, missing outcome data, measurement of the outcome, and selection of the reported result (ranging from 0.81 to 0.90).

### Pain

**FIGURE 3:**
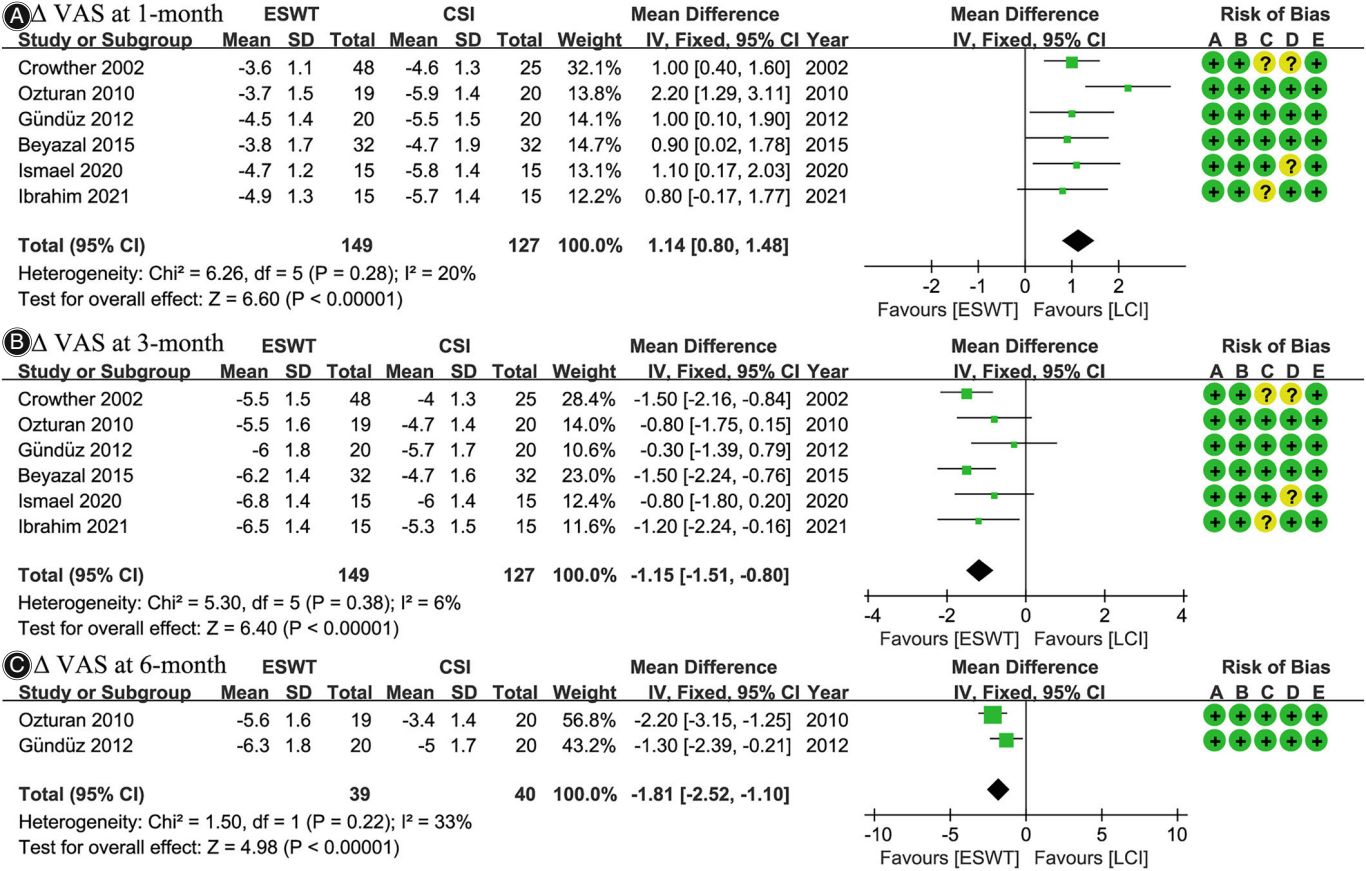
Meta‐analysis of change in visual analogue scale (Δ VAS): (A) at 1‐month follow‐up; (B) at 3‐month follow‐up; and (C) at 6‐month follow‐up. The green squares represent the effect estimate of the individual studies and the horizontal lines indicate the confidence interval, and the dimension of the square reflects the weight of each study. The black diamond represents the combined point estimate and confidence intervals. The risk of bias, categorized from A to E, is consistent with the order of the RoB2 items presented in Figure 2.

All six studies^21^, ^22^, ^23^, ^24^, ^25^, ^26^ reported Δ VAS at 1‐month and 3‐month follow‐ups. A significantly greater change in Δ VAS was observed in favor of LCI at the 1‐month follow‐up (WMD, 1.14; 95% CI, 0.80 to 1.48; *I* ^2^ = 20%; *p <* 0.001), while at the 3‐month follow‐up, it favored ESWT (WMD, −1.15; 95% CI, −1.51 to −0.80; *I* ^2^ = 6%; *p <* 0.001). Two studies^23^, ^26^ reported Δ VAS at 6‐month follow‐up, indicating that ESWT resulted in a greater reduction in VAS (WMD, −1.81; 95% CI, −2.52 to −1.10; *I* ^2^ = 33%; *p <* 0.001) (Figure 3).

### Grip Strength

**FIGURE 4:**
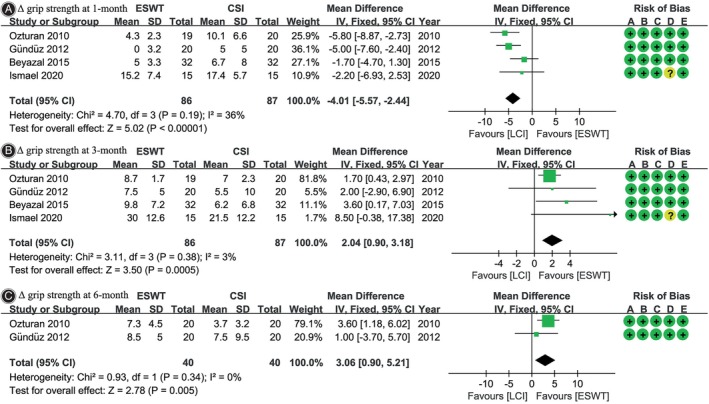
Meta‐analysis of Δ grip strength: (A) at 1‐month follow‐up; (B) at 3‐month follow‐up; and (C) at 6‐month follow‐up. The green squares represent the effect estimate of the individual studies and the horizontal lines indicate the confidence interval, and the dimension of the square reflects the weight of each study. The black diamond represents the combined point estimate and confidence intervals. The risk of bias, categorized from A to E, is consistent with the order of the RoB2 items presented in Figure 2.

Four^21^, ^23^, ^25^, ^26^ out of six studies reported Δ grip strength at 1‐month and 3‐month follow‐ups. At 1‐month follow‐up, the LCI group exhibited a greater Δ grip strength (WMD, −4.01; 95% CI, −5.57 to −2.44; *I* ^2^ = 36%; *p <* 0.001), whereas at 3 months, the ESWT group showed a greater Δ grip strength (WMD, 2.04; 95% CI, 0.90 to 3.18; *I* ^2^ = 3%; *p* = 0.0005). Two studies^23^, ^26^ reported Δ grip strength at 6‐month follow‐up, indicating that ESWT resulted in a greater improvement in grip strength (WMD, 3.06; 95% CI, 0.90 to 5.21; *I* ^2^ = 0%; *p* = 0.005) (Figure 4).

### PRTEE Score

**FIGURE 5:**
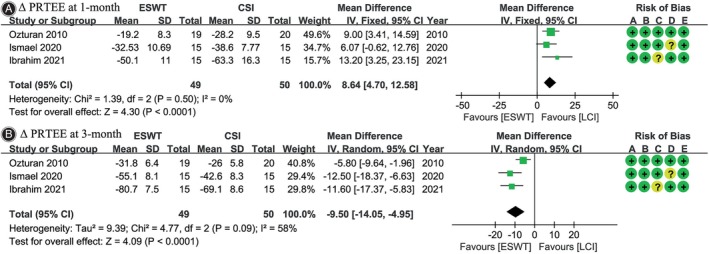
Meta‐analysis of change in patient‐rated tennis elbow evaluation (Δ PRTEE) score: (A) at 1‐month follow‐up; (B) at 3‐month follow‐up. The green squares represent the effect estimate of the individual studies and the horizontal lines indicate the confidence interval, and the dimension of the square reflects the weight of each study. The black diamond represents the combined point estimate and confidence intervals. The risk of bias, categorized from A to E, is consistent with the order of the RoB2 items presented in Figure 2.

Three^24^, ^25^, ^26^ out of six studies reported Δ PRTEE scores at 1‐month and 3‐month follow‐ups. At the 1‐month follow‐up, the LCI group demonstrated larger Δ PRTEE scores (WMD, 8.64; 95% CI, 4.70 to 12.58; *I* ^2^ = 0%; *p <* 0.001), while at 3 months, the ESWT group exhibited greater Δ PRTEE scores (WMD, −9.50; 95% CI, −14.05 to −4.95; *I* ^2^ = 58%; *p <* 0.001) (Figure 5).

### Adverse Events

**FIGURE 6:**
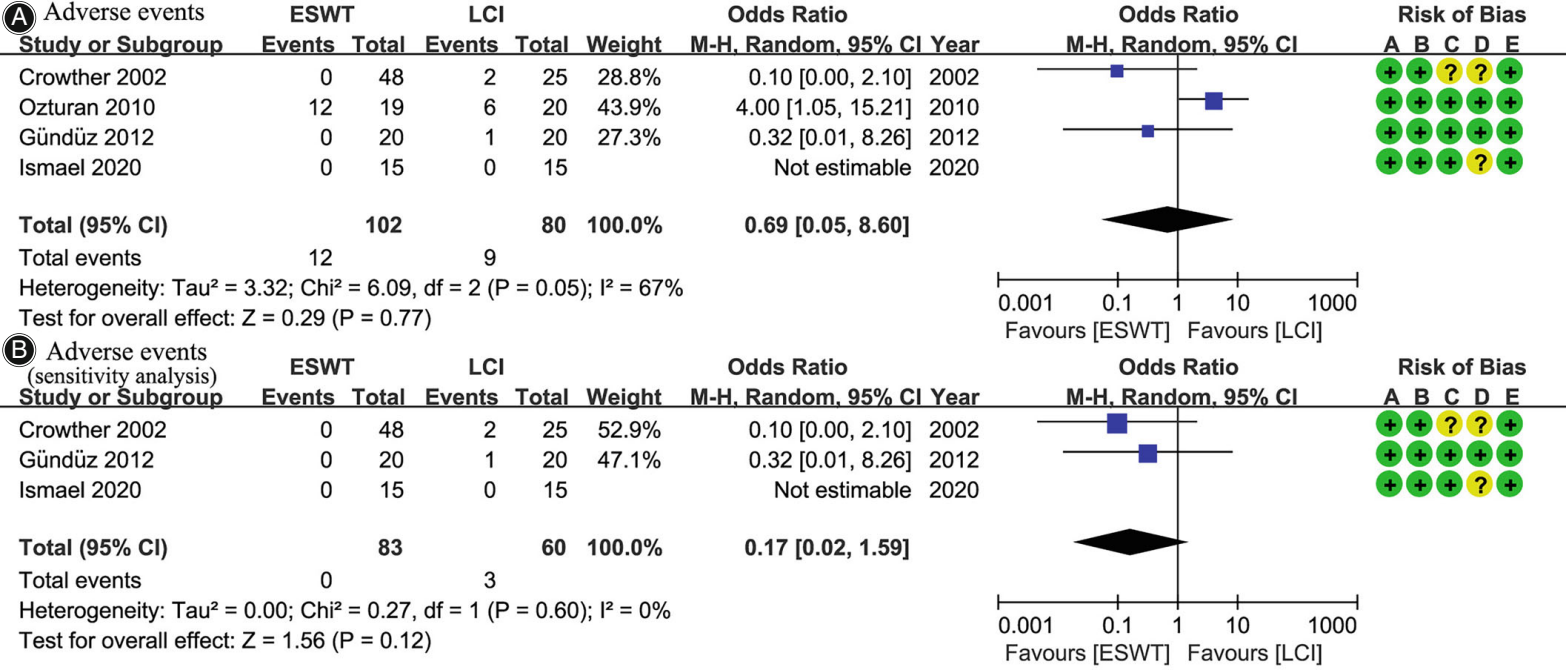
Meta‐analysis of adverse events: (A) original analysis; (B) sensitivity analysis. The blue squares represent the effect estimate of the individual studies and the horizontal lines indicate the confidence interval and the dimension of the square reflects the weight of each study. The black diamond represents the combined point estimate and confidence intervals. The risk of bias, categorized from A to E, is consistent with the order of the RoB2 items presented in Figure 2.

Four^22^, ^23^, ^25^, ^26^ out of six studies reported adverse events following ESWT or LCI. The two interventions displayed a similar incidence of adverse events (OR, 0.69; 95% CI, 0.05 to 8.60; *I* ^2^ = 67%; *p* = 0.77), all of which were mild (Figure 6A). These adverse events typically manifest as discomfort, inconvenience, or temporary limitations, such as swelling, bruising, or irritation around the intervention site. However, they generally resolve spontaneously over time or with minimal intervention.

Compared with other included studies, Ozturan et al.^26^ reported a significantly higher number of adverse events, because they defined transient pain after ESWT or LCI treatment as an adverse event. To mitigate its potential impact, we performed a sensitivity analysis by excluding this study, which still showed no significant difference in adverse events between the two groups (OR, 0.17; 95% CI, 0.02 to 1.59; *I* ^2^ = 0%; *p* = 0.12) (Figure 6B).

## Discussion

This study, comprising 6 RCTs and involving 276 patients, demonstrates the effectiveness and safety of both ESWT and LCI in treating chronic LE. Both interventions displayed a similar incidence of adverse events, all of which were mild. Notably, while ESWT displayed inferior short‐term (1‐month follow‐up) outcomes, it showed superior long‐term (3‐month and 6‐month follow‐ups) effectiveness in alleviating pain, enhancing grip strength, and improving PRTEE scores compared with LCI.

### Comparison with Previous Studies

To our best knowledge, this systematic review with meta‐analysis is the most current and highest LOE available on the comparison of ESWT and LCI in treating chronic LE. Two recent meta‐analyses^27^, ^28^ of RCTs comparing ESWT with other treatments for LE yielded inconsistent results. Yao et al.^27^ concluded that ESWT is effective in treating LE and boasts better overall safety compared with several other methods. However, Zheng et al.^28^ concluded that while ESWT may not effectively reduce mean overall pain, it demonstrates greater success in achieving a 50% pain reduction, suggesting it may be a preferable option for LE treatment. Another systematic review and network meta‐analysis,^29^ which incorporated RCTs to compare ESWT with various injections, concluded that DPT and ESWT emerged as the top two treatment options for pain control. Additionally, ESWT was identified as the optimal choice for grip strength recovery, whereas CSs were not recommended for the treatment of LE. It is worth noting that these prior meta‐analyses included studies comparing ESWT with various control groups, with most not directly comparing ESWT and LCI head‐to‐head.

The only meta‐analysis^30^ that reported a head‐to‐head comparison of ESWT and LCI included four RCTs. This study revealed significant differences in VAS score and grip strength between the two groups, leading to the conclusion that ESWT may represent a superior alternative for treating LE. However, this study only presented VAS score and grip strength assessed at a single extended follow‐up point, thereby lacking comparisons of short‐term outcomes and safety. In contrast, our study offers a more comprehensive perspective by encompassing additional RCTs and reporting changes in VAS, grip strength, PRTEE scores, and adverse events across multiple time points. Our findings indicate that LCI yielded superior short‐term outcomes compared with ESWT for chronic LE, a result not previously reported by any prior systematic review or meta‐analysis.

### Interpretation of Outcomes

The mechanism of each treatment modality potentially contributes to the observed differences in short‐term and long‐term outcomes. LCI provides rapid symptom relief by directly targeting inflammation and pain through the delivery of CSs to the affected area, using the potent anti‐inflammatory properties of CSs.^31^, ^32^, ^33^ However, the effects of a single LCI can only last from a few weeks to several months.^31^ Some evidence suggests that long‐term outcomes may be worse in terms of pain and function compared with a wait‐and‐see approach.^34^, ^35^ In an RCT^36^ with 185 participants, assessing LCI, physiotherapy, and a wait‐and‐see approach in primary care, success rates at 6 weeks were 92%, 47%, and 32%, respectively. However, by 52 weeks, success rates declined to 69%, 91%, and 83%, respectively. Notably, while LCI initially demonstrated the highest success rate at 6 weeks among the interventions, it showed the lowest success rate at 52 weeks. Histologic studies demonstrating decreased collagen production and fibroblast viability in tendon tissue after a single LCI suggests a potential mechanism for adverse outcomes.^37^ While some individuals use repeated injections to sustain long‐term effectiveness, these carry an increased risk of skin thinning, atrophy, and tendon degeneration.^38^ Notably, in this analysis, all included studies administered only a single LCI, with no such adverse events reported.

In contrast, ESWT is an effective method, distinguished by its noninvasive nature, ease of application, and high tolerance among the majority of patients with chronic LE, particularly those who have not responded to initial conservative treatments.^39^, ^40^ While its exact mechanism is not fully understood, it is believed that ESWT induces tissue regeneration and remodeling by promoting angiogenesis, cellular proliferation, extracellular matrix synthesis, and inhibiting pain receptors.^41^ As tissue healing effects typically begin around 1 month after ESWT,^42^ the initial outcomes may theoretically be inferior to those of LCI. However, the gradual improvement in tissue quality often result in more sustained and long‐lasting improvements in pain and function. Our findings support this potential mechanism, as ESWT demonstrated significantly superior pain relief, grip strength recovery, and reduction in PRTEE scores compared with LCI from the 12‐week follow‐up onward.

In this study, we found both two interventions had a similarly low rate of mild adverse events. The rates of adverse events were 11.8% (12 of 102) in the ESWT group, and 11.3% (9 of 80) in the LCI group. Due to Ozturan et al.'s^26^ broader definition (transient pain after treatment) of adverse events, we conducted a sensitivity analysis by excluding their study, which showed low adverse event rates of 0% (0 of 83) for ESWT and 5% (3 of 60) for LCI, with no significant difference between the two groups (*p* = 0.12). The adverse events observed in the ESWT group included temporary pain, nausea, swelling, or local erythema at the elbow, as described in previous studies.^43^, ^44^ Within the LCI group, adverse events primarily consisted of pain and discoloration at the injection site, with no instances of infection or other serious adverse events reported. It is worth noting that all the included studies utilized ESWT for at least three sessions, whereas the LCI group received only one injection. Interestingly, the repeated sessions of ESWT did not result in an increase in adverse events, highlighting its well‐tolerated and repeatable nature. This may represent another advantage of ESWT over LCI.

### Limitations and Strengths

The study has some limitations. First, the number of available studies was limited due to the restriction of a head‐to‐head RCT design in the literature. Second, despite well‐matched patient groups in both interventions, differences in crucial factors such as sports level, severity of LE, ESWT equipment specifications, LCI composition, rehabilitation protocols, and follow‐up durations across studies were inevitable. Third, previous studies have indicated that the type of ESWT may influence the outcomes of treating LE.^14^ However, a subgroup analysis to separately compare radial or focused ESWT and LCI was not conducted in our study due to the limited sample sizes available. Last, only two of the included studies reported outcomes beyond 6 months, leaving results at longer follow‐ups absent. Consequently, outcomes that can only be observed at long‐term follow‐up, such as the tendon assessment, remain absent in this study.

The study's strength is underscored by its comprehensive approach to data synthesis, meticulous quality assessment of the included RCTs, and the incorporation of the latest research findings. This up‐to‐date synthesis of evidence provides valuable insights applicable to clinical practice. The principal findings of our research carry profound clinical implications, serving as a guide for healthcare providers in tailoring treatment strategies to align with individual patient preferences for immediate or sustained symptom relief.

## Conclusion

Both ESWT and LCI are effective and safe in treating chronic LE. Compared with LCI, ESWT showed inferior short‐term (1‐month) but superior long‐term (3‐month and 6‐month) outcomes regarding pain relief and function recovery, with a similar rate of mild adverse events. Long‐term studies with extended follow‐ups are essential for assessing the sustainability of treatment effects and detecting potential late complications. Moreover, investigating the optimal ESWT parameters and the influence of patient‐specific factors could lead to more personalized and effective treatment strategies.

## Author Contributions

L.Z. conceptualized the study, developed the methodology, and handled the software aspects. L.Z. and X.Z. were responsible for the methodology and software. Formal analysis and data curation were conducted by L.Z. and L.P. L.Z. prepared the original draft, while X.Z., L.P., Z.W., and J.J. reviewed and edited it. Visualization was done by L.Z. and L.P., and supervision was provided by J.J.

## Conflict of Interest

The authors declare no conflicts of interest.

## Funding Information

This study was supported by the Postdoctoral Research Fund of West China Hospital, Sichuan University No. 2023HXBH078.
